# Indispensable Role of HIF-1α Signaling in Post-implantation Survival and Angio-/Vasculogenic Properties of SHED

**DOI:** 10.3389/fcell.2021.655073

**Published:** 2021-07-23

**Authors:** Yuanyuan Han, Qixin Chen, Lili Zhang, Waruna Lakmal Dissanayaka

**Affiliations:** Applied Oral Sciences & Community Dental Care, Faculty of Dentistry, The University of Hong Kong, Pokfulam, Hong Kong

**Keywords:** HIF-1α, post-implantation survival, cell metabolism, redox balance, regenerative medicine, dental stem cells

## Abstract

**Objectives:**

Post-implantation survival and timely vascularization of stem-cell based constructs are critical factors in achieving successful outcomes in tissue regeneration approaches. Hypoxia inducible factor-1α (HIF-1α) is known to mediate adaptive functions to ischemic stress in many different cell types. The current study aimed to explore the role of HIF-1α in post-implantation survival and angio-/vasculogenesis of stem cells from human exfoliated deciduous teeth (SHED).

**Methods:**

HIF-1α in SHED was suppressed using siRNA or chemical inhibitor (YC-1) and used in Matrigel plug assay conducted on severe combined immunodeficient mice. The plugs were retrieved on day 3 or 7 post-injection and analyzed for hypoxia status, ki67 expression, DNA fragmentation (TUNEL), cellularity, and vascularization by histology and immunohistochemistry for CD31, HIF-1α, pyruvate dehydrogenase kinase-1 (PDK1), hexokinase 2 (HK2), and glucose transporter 1 (Glut1). Cell viability of HIF-1α silenced SHED under different stress conditions (hypoxia, H_2_O_2_, and low glucose) *in vitro* was measured by CCK-8 assay. CM-H_2_DCFDA and MitoSOX Red were used to detect cellular and mitochondrial reactive oxygen species (ROS) levels, respectively. PDK1, HK2, and Glut1 expression were measured by western blotting and immunofluorescence. Secretory protein levels of vascular endothelial growth factor (VEGF) and the respective paracrine effects on endothelial cell proliferation and migration were detected by ELISA, CCK-8 assay, and *trans*-well assay, respectively.

**Results:**

Histological analysis of Matrigel plugs showed significantly reduced cell survival in HIF-1α silenced or chemically inhibited SHED groups, which could be attributed to diminished metabolic adaptations as shown by decreased PDK1, HK2, and Glut1 expression. HIF-1α inhibition in SHED also resulted in significantly low blood vessel formation as observed by a low number of perfused and non-perfused vessels of human or mouse CD31 origin. The viability of HIF-1α silenced SHED was significantly affected under hypoxia, H_2_O_2_, and low-glucose conditions *in vitro*, which was reflected in increased cytoplasmic and mitochondrial ROS levels. Significantly reduced levels of VEGF in HIF-1α silenced SHED resulted in decreased paracrine angiogenic effects as shown by low proliferation and migration of endothelial cells.

**Conclusion:**

HIF-1α plays an indispensable role in post-implantation survival and angio-/vasculogenic properties of SHED by maintaining ROS homeostasis, inducing metabolic adaptations, and VEGF secretion.

## Introduction

Stem-cell-based tissue engineering is a promising approach to restore damaged, injured, or missing tissues, in which stem cells differentiate into specific phenotypes and induce a regenerative microenvironment (Caplan, 2007). Stem cells from human exfoliated deciduous teeth (SHED) are regarded as one of the most striking cell sources because of their easy isolation, highly self-renew ability, and multiple differentiation capacities (Han et al., 2020b). There are, however, two critical challenges for stem-cell-based tissue engineering to overcome in order to achieve consistent preclinical and clinical trial outcomes. One major challenge is the low post-implantation cell survival, which can vary from as low as 10% up to 60% (Becquart et al., 2012; Stegen et al., 2016). Once the cells are introduced into the *in vivo* microenvironment, they experience an immediate hostile condition where oxygen and nutrient supply are scarce and oxidative stress is high, which can lead to significant cell death (Becquart et al., 2012). The second challenge is to achieve timely vascularization of the engineering construct in order to secure a constant supply of nutrients and oxygen, and ensure the removal of cell metabolic waste, which is vital for the long-term survival and functionality of the regenerating tissues.

Cells have developed physiological adaptive mechanisms for hypoxic/ischemic stress, which are mainly mediated through hypoxia-inducible factors (HIFs). HIF-1α, a master transcription factor in hypoxia, is specifically stabilized under low oxygen conditions as oxygen acts as a cofactor for prolyl hydroxylases that initiate degradation of HIF-1α. In hypoxia, HIF-1α interacts with ubiquitously expressed HIF-1β, and activates the expression of target genes containing hypoxia response elements (Rashid et al., 2019). Cells produce reactive oxygen species (ROS) as a byproduct of the electron transport chain, which is essential in cellular energy production; however, in hypoxia, severe increase of ROS will lead to cell death (Zhang et al., 2019). Once the cellular redox homeostasis is disrupted, oxidative stress in the cell may lead to DNA/RNA damage, contributing to cell death (Zhang et al., 2020; Chen et al., 2021). HIF-1α signaling pathway is found to be tightly interconnected with the ROS pathway. Hypoxia-inducible ROS could contribute to HIF-1α accumulation, and increased HIF-1α could prevent ROS overproduction (Wheaton and Chandel, 2011). The major target of HIF-1α on decreasing endogenous ROS is through pyruvate conversion to acetyl-CoA by pyruvate dehydrogenase kinase 1 (PDK1), which activates the tricarboxylic acid (TCA) cycle enzymes (Kim et al., 2006).

Cell survival and cellular metabolism are also closely related since tissue repair is an energy-demanding process that includes cell proliferation, self-renewal, and cell differentiation. Metabolic pathways, however, are very sensitive to cellular cues, such as oxygen tension and, in general, triggered by hypoxia. It is reported that HIF-1α has the function of regulating glucose metabolites by switching mitochondrial respiration to cytosolic glycolysis in cancer cells (Lu et al., 2002). Accordingly, under hypoxia, HIF-1α stabilization and formation of HIF-1α/β complex activate genes encoding a series of glycolytic enzymes and glucose transporters (Zhu et al., 2014).

In addition to metabolic adaptations, HIF-1α expression is known to induce an angiogenic response through vascular endothelial growth factor (VEGF; Ahluwalia and Tarnawski, 2012). VEGF plays an essential role in activating cellular pathways that lead to the promotion of endothelial cell proliferation, migration and assembly into vascular structures (Kliche and Waltenberger, 2001). Therefore, upregulation of VEGF-VEGFR signaling pathways under hypoxia assists the recovery of cells or tissues affected by ischemia (Ramakrishnan et al., 2014).

Despite the reported functions of HIF-1α in relation to other cell types, its role and mechanisms on post-implantation cell survival and angio-/vasculogenic properties of dental stem cells under stress conditions are still not clear. In the current study, we aimed to investigate the significance of HIF-1α expression in SHED in cell survival and angio-/vasculogenesis using an *in vivo* Matrigel plug assay. We found that genetic silencing or chemical inhibition of HIF-1α in SHED reduces the cell survival and angio-/vasculogenic response significantly. Furthermore, we revealed that HIF-1α regulates the expression of target proteins that mediate adaptive metabolic mechanisms in both *in vivo* and *in vitro* survival and angiogenesis in SHED.

## Materials and Methods

### Cell Culture

SHED were purchased from AllCells (Alameda, CA, United States) and cultured in α-Modified Eagle’s Medium (α-MEM) supplemented with 10% FBS and 1% penicillin/streptomycin. Mesenchymal origin and multipotent differentiation capacity of the cells were evaluated and published in our previous study (Xu et al., 2017). Human umbilical vein endothelial cells (HUVECs) were purchased from ScienCell (Carlsbad, CA, United States) and cultured in endothelial growth medium-2 (EGM-2, Lonza, Walkersville, MD, United States). All cell cultures were kept in a 37°C and 5% CO_2_ incubator. Passage 4–7 of SHED and 3–6 of HUVECs were used in all the downstream experiments. Hypoxia condition (1% O_2_) *in vitro* cultures was achieved using the hypoxia incubator (Thermo Scientific, MA, United States).

### HIF-1α Knockdown by siRNA and Chemical Inhibition by YC-1

Silencing of HIF-1α expression in SHED was achieved by transfection of premade siRNA (siHIF-α:4390824, negative control: 4390843, Thermo Scientific) with Lipofectamine^TM^ 3000 (Thermo Scientific) according to the manufacturer’s instructions. Briefly, the cells were seeded in 6-well plates for 70 ∼ 90% density. After overnight incubation, lipofectamine^TM^ 3000 reagent – siRNA complexes were prepared in Opti-MEM^TM^ Reduced Serum Medium (Thermo Scientific). Subsequently, the fresh culture medium was changed and siRNA-lipid complexes were added into the cells. siRNA carrying a no significant sequence was used as a negative control. Transfection efficiency was assessed by western blotting (WB) and immunofluorescence (IF) after 48 h under hypoxia. For identification of different groups, we termed the siRNA-control group as “siControl” and the siRNA-HIF-1α group as “siHIF-1α.” To confirm the function of HIF-1α, we also used HIF-1α inhibitor YC-1 (80 μM, Sigma, MO, United States), which acts by prevention of HIF-1α accumulation in response to hypoxia. Accordingly, we termed the YC-1-control and YC-1-treated groups as “CTR” and “YC-1” groups, respectively.

### *In vivo* Matrigel Plug Assay

All experimental animal procedures were approved by the Committee on the Use of Live Animals in Teaching and Research (CULATR 4625-18) of the University of Hong Kong and performed following the Guide for the Care and Use of Laboratory Animals published by the United States National Research Council and regulations. 24 severe combined immunodeficient (SCID/CB17) mice (male, ∼6 weeks of age, 20 ∼ 40 g) were randomly allocated into eight groups: siControl-3 days, siHIF-1α-3 days, siControl-7 days, siHIF-1α-7 days, CTR-3 days, YC-1-3 days, CTR-7 days, and YC-1-7 days. Each group had 3 mice (6 plugs per group, *n* = 6). A subcutaneous injection procedure was performed as described before (Han et al., 2020a). Briefly, the cells (3 × 10^6^/per plug, cells were treated with siRNA *in vitro* for 48 h) were trypsinized, centrifuged, and resuspended in 100 μL EGM-2 followed by mixing with 400 μL of Matrigel and injected slowly into the subcutaneous space of the lateral hind regions of mice bilaterally. In the chemical inhibition group, YC-1 dissolved in DMSO was mixed with cell/Matrigel solution to achieve a final concentration of 80 μM before injection. Matrigel plugs were retrieved after 3 days (3 D) and 7 days (7 D) of injection, fixed with 4% paraformaldehyde (PFA) for 24 h and paraffin-embedded in an orientation that allowed obtaining sections with intact skin and muscle layer. Sections of 5 μm thickness were cut and examined for histology and immunohistochemistry (IHC).

### Histology and Immunohistochemistry

Histological staining (H&E), IHC, and quantification procedures were performed as described previously (Han et al., 2020a). For H&E staining, sections were immersed in hematoxylin for 3 min and eosin for 1 min after alcohol gradient dewaxing. After mounting, images were taken with an inverted microscope (Nikon Eclipse LV100N POL, Tokyo, Japan) at 20× and 50× magnifications. Vessel-like structures without red blood cells were counted as non-perfused vessels and structures with red blood cells in the lumen were counted as perfused vessels. For IHC staining, following primary antibodies were used: hypoxyprobe omni kit (Hypoxyprobe, MA, United States), ki67 (ab92742, Abcam), isotype control (ab37415, Abcam), human CD31 (ab32457, Abcam), mouse CD31 (ab124432, Abcam), HIF-1α (ab51608, Abcam), PDK1 (sc-293160, Santa Cruz, TX, United States), hexokinase 2 (HK2, sc-374091, Santa Cruz), and glucose transporter 1 (Glut1, ab150299, Abcam). Mouse and rabbit specific HRP/DAB IHC detection kit (Abcam) was used according to the protocol. Briefly, following the deparaffinization process, sections were incubated with hydrogen peroxide block for 10 min, antigen retrieval pretreatment for 6 min, protein block for 5 min, and different primary antibodies overnight. On the following day, sections were incubated with anti-mouse and rabbit secondary antibody for 10 min, streptavidin peroxidase for 10 min, and DAB solution for 10 s – 1 min. Lastly, sections were counterstained with hematoxylin for 1 min. After capturing images by NIS-Elements AR 3.1 software (Nikon, Tokyo, Japan) at 20× and 50× magnification, percentages of positive areas/cell numbers (HIF-1α, PDK1, HK2, Glut1, and human CD31) were calculated on 5 randomly selected regions under 50 × magnification using NIS-Elements AR 3.1 software (Nikon, Tokyo, Japan). *In situ* direct DNA fragmentation assay (TUNEL Assay Kit, Abcam) was performed following the kit protocol to visualize the DNA damage in tissue. Fluorescent microscope (Nikon, Tokyo, Japan) was used to capture the TUNEL-positive cells.

### Conditioned Media

siControl and siHIF-1α SHED were cultured in standard culture medium until 80% confluence and changed to serum-free medium. Conditioned media were collected after 24 h in normoxia and hypoxia condition, followed by centrifugation at 1,500 rpm and filtration to remove cell debris.

### CCK-8 Assay

#### SHED in Stress Condition CCK-8 Assay

To analyze *in vitro* stress resistance, siControl and siHIF-1α SHED were cultured for 24–48 h in the presence of either hypoxia, hydrogen peroxide (H_2_O_2_, 200 μM), glucose-deprived medium, or hypoxia + H_2_O_2_ + glucose-deprived medium condition. *In vitro* cell viability was detected by CCK-8 assay kit (Abcam).

#### HUVECs in Conditioned Media CCK-8 Assay

To examine the paracrine effects of siHIF-1α SHED on the proliferation of endothelial cells, HUVECs were seeded on 96-well plates at a density of 6,000 cells per well. After 24 h, EGM-2 (positive control), α-MEM (negative control), conditioned media from siControl or siHIF-1α SHED in normoxia or hypoxia were added to separate wells. After culturing for 48 h, cell proliferation was determined using the CCK-8 assay kit. Briefly, fresh medium was changed and 10 μl CCK-8 reagent was added per well. After incubating for 3 h at 37°C, the absorbance was measured at 460 nm by SpectraMax^®^ M2 microplate reader (Molecular Devices, CA, United States).

### Western Blotting and Immunofluorescence

Western blotting and IF were performed to evaluate the specific protein expression as described previously (Han et al., 2020a). In addition to the above-mentioned primary antibodies for IHC staining, HIF-1α (610958, BD Biosciences, New Jersey, United States) for WB was also used. Secondary antibodies included anti-rabbit or anti-mouse HRP-linked antibody (Cell Signaling Technology, MA, United States), Alexa Fluor 488^®^-conjugated goat anti-mouse antibody (Abcam), Alexa Fluor 594^®^-conjugated goat anti-rabbit antibody (Abcam). The protein samples of siControl and siHIF-1α SHED were collected after culturing under normoxia and hypoxia for 24 h. The results of WB were quantified by Image J and normalized by Glyceraldehyde 3-phosphate dehydrogenase (GAPDH). Cells seeded on coverslips in 24-well plates were fixed by 4% PFA after incubated in normoxia and hypoxia for IF staining. After incubated with primary antibody overnight, secondary antibody for 1 h and DAPI for 5 min, images were captured by fluorescence microscope (Nikon, Tokyo, Japan) at 20 × magnification.

### Detection of Cellular ROS Levels

CM-H_2_DCFDA (Thermo Scientific) and MitoSOX Red (Thermo Scientific) staining were used for detecting cytoplasmic and mitochondrial ROS levels, respectively. Cells were seeded in 24-well plates and cultured in normoxia or hypoxia for 24 h. Subsequently, cells were incubated with 10 μM CM-H_2_DCFDA for 30 min or 5 μM MitoSOX Red for 10 min at 37°C, and fluorescence was captured by immunofluorescence microscope (Nikon, Tokyo, Japan) at 20 × magnification. Immunofluorescence density was quantified by Image J software (U. S. National Institutes of Health, MD, United States) and normalized by the siControl normoxia group.

### Cell Migration Assay

*Trans*-well assay was performed to study the paracrine effects of siHIF-1α SHED on endothelial cells as described before (Han et al., 2020a). Briefly, HUVECs were trypsinized, resuspended with serum-free medium and seeded on the upper chamber of inserts at a density of 1 × 10^4^/200 μL, whereas 600 μL of conditioned media from siControl or siHIF-1α SHED were added to the lower compartment of each well. After 24 h, HUVECs on the lower surface of the membrane were fixed and stained with 0.1% (w/v) crystal violet (Sigma) for 15 min. Migrated HUVEC numbers were quantified.

### ELISA

VEGF secretion levels were measured in the culture supernatant of siHIF-1α SHED and siControl SHED using the human VEGF DuoSet Elisa Kit (R&D systems, MN, United States) as described before (Han et al., 2020a). Supernatants were collected from siControl and siHIF-1α SHED after culturing in normoxic and hypoxic conditions for 24 h. Protein levels were normalized to that of siControl normoxia group.

### Statistical Analysis

All the experiments were conducted in triplicate and repeated at least 3 times. Results were presented as mean ± standard deviation (SD). Statistical analysis was carried out using Student’s *t*-test for assays done between two groups, and one-way ANOVA with a Tukey’s *post hoc* test in multiple comparisons. Two-way ANOVA with Sidak Test in multiple comparisons was used for two independent variables on a dependent variable. All the statistical testing and creation of graphs were performed using Prism 8.0 software (GraphPad Software, Inc., San Diego, CA, United States). Statistical significance was considered at *p* < 0.05.

## Results

### Silencing of HIF-1α Impairs the Post-Implantation Cell Viability in Matrigel Plugs

**FIGURE 1:**
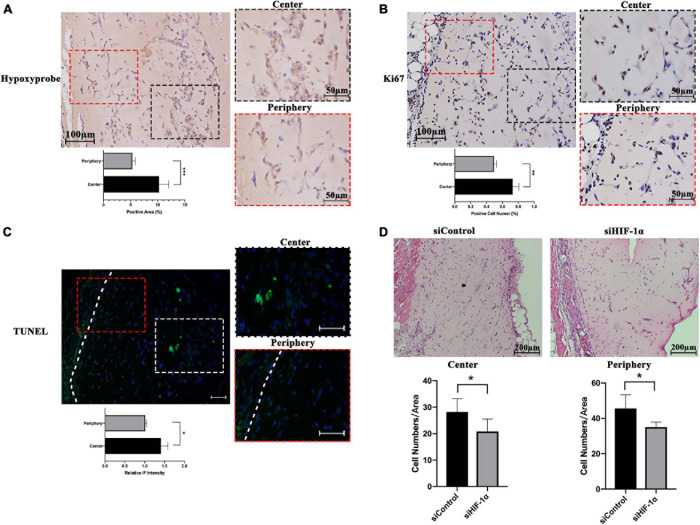
Hypoxic status and cell survival within SHED-seeded Matrigel plugs *in vivo*. Immunohistochemistry for **(A)** Hypoxyprobe and **(B)** ki67, and **(C)** immunofluorescence for TUNEL assay in the center and the periphery of the Matrigel plug **(D)**. Cellularity at the center and the periphery of H&E stained Matrigel plugs of siControl and siHIF-1α SHED after 7 days of implantation. White dotted line – the boundary between Matrigel plug and mouse tissue. *p < 0.05, **p < 0.01, and ***p < 0.001. Scale bar: 10 × = 200 μm, 20 × = 100 μm, 50 × = 50 μm, and IF = 50 μm.

The hypoxic status of the implanted Matrigel plugs was investigated by IHC for Hypoxyprobe^TM^ (pimonidazole hydrochloride), which is a 2-nitroimidazole that is activated specifically in hypoxic cells. SHED-seeded Matrigel plugs showed significantly higher number of hypoxic cells in the central region of the construct as evident by positive IHC staining (Figure 1A). Cell proliferation and apoptosis in hypoxia *in vivo* were evaluated by IHC for Ki67, a proliferative marker (Figure 1B) and DNA fragments (TUNEL) assay (Figure 1C), respectively. Higher number of cells with ki67 expression and DNA fragments were found in the center of the Matrigel compared with that of the periphery, which means the cell viability under hypoxia is a result of countervailing effects between cell apoptosis and cell proliferation. Accordingly, as shown by H&E staining and quantification of cellularity after 7 days (Figure 1D), both the central and peripheral areas of the siHIF-1α plug contained significantly less number of survived cells compared with that of the siControl group.

### Silencing or Chemical Inhibition of HIF-1α Down-Regulated the Proteins Important in Metabolic Adaptations *in vivo*

**FIGURE 2:**
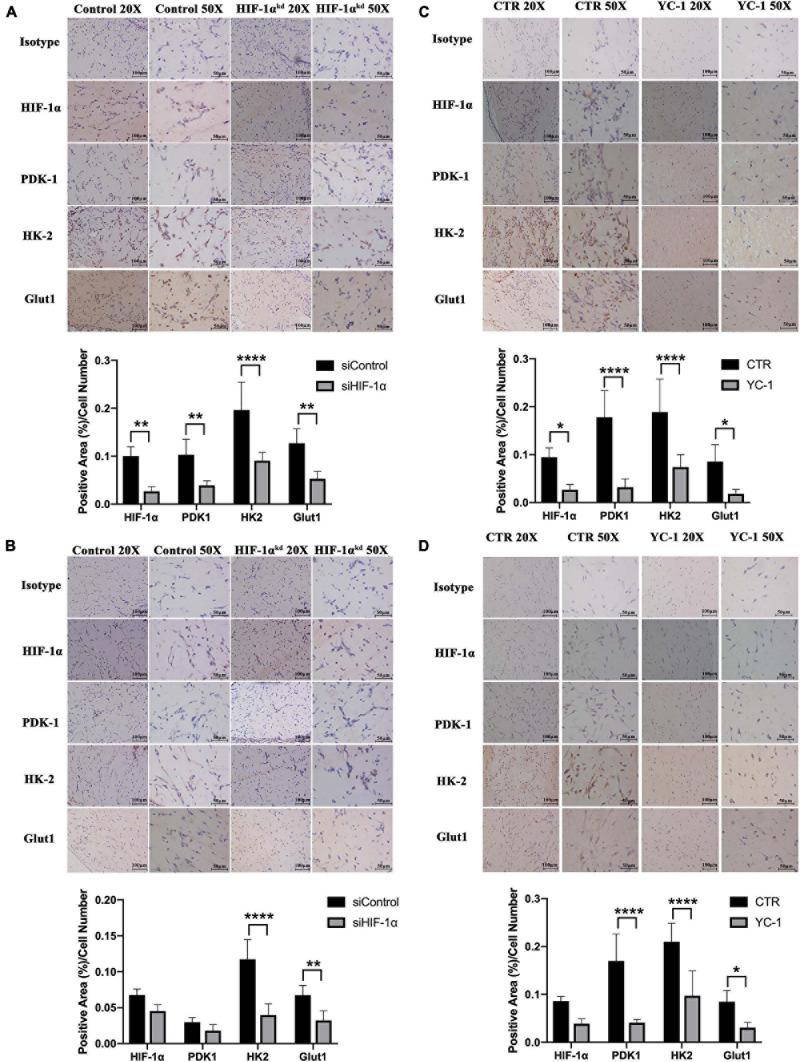
Silencing or chemical inhibition of HIF-1α down-regulated the proteins important in metabolic adaptations *in vivo*. Immunohistochemistry for HIF-1α, PDK-1, HK-2, and Glut-1 in siControl and si-HIF-1α SHED containing Matrigel plugs at **(A)** 3 days and **(B)** 7 days, and CTR and YC-1 SHED containing groups at **(C)** 3 days and **(D)** 7 days of transplantation. *p < 0.05, **p < 0.01, and ****p < 0.0001. Scale bar: 20 × = 100 μm, 50 × = 50 μm.

It has been reported that in severely hypoxic conditions, HIF-1α-mediated induction of genes could regulate metabolic shift from oxidative phosphorylation to glucose oxidation by preventing the entry of pyruvate into the tricarboxylic cycle. To investigate the role of HIF-1α signaling in maintaining early post-implantation cell survival, we immunohistochemically stained the tissue sections for the expression of proteins important in the metabolic shift to glycolysis as well as regulating cellular ROS homeostasis. As shown in Figure 2A (3 D) and Figure 2B (7 D), the siHIF-1α group demonstrated less PDK1 (*p* < 0.01), HK2 (*p* < 0.0001), and Glut1 expression (*p* < 0.01) compared with the control groups. Similarly, as shown in Figure 2C (3 D) and Figure 2D (7 D), chemical inhibition of HIF-1α by YC-1 significantly suppressed the expression of PDK1, HK2, and Glut1 at both 3 D and 7 D of transplantation. When compared with 7 D samples, we observed that the expression of these proteins were higher in 3 D plugs, which indicated a more critical role at the early stages of post-implantation.

### Silencing or Inhibition of HIF-1α Impairs the Angio-/Vasculogenic Capacity of SHED *in vivo*

**FIGURE 3:**
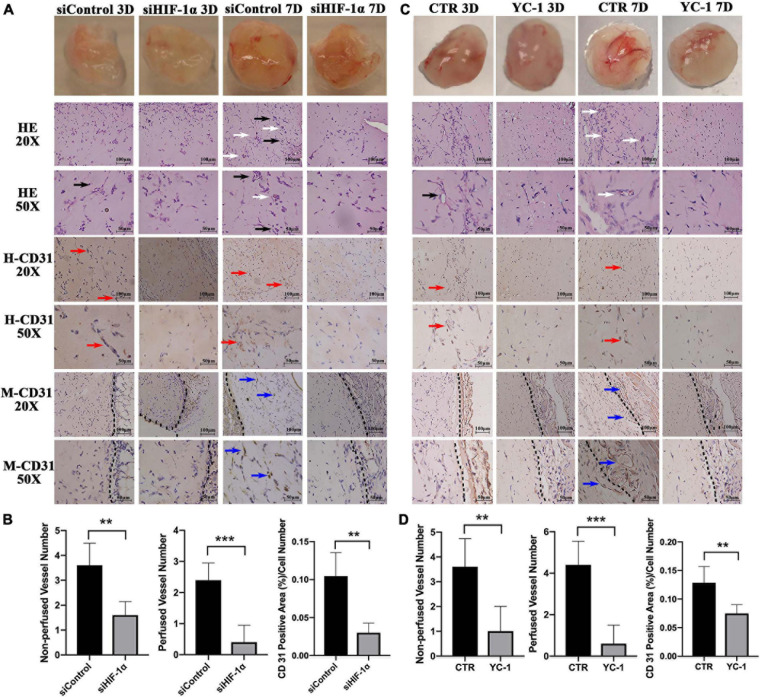
Silencing or chemical inhibition of HIF-1α expression impairs the angio-/vasculogenic capacity of SHED. Macroscopic photos, H&E staining, immunohistochemical staining for human CD31 and mouse CD31 of **(A)** siControl SHED and siHIF-1α SHED **(C)** CTR SHED and YC-1 SHED Matrigel plugs 3 days and 7 days after implantation. Quantified graphs of non-perfused and perfused vessel numbers and percentages of human CD31 positive area of **(B)** siControl SHED and siHIF-1α SHED **(D)** CTR SHED and YC-1 SHED Matrigel plugs at 7 days after implantation. Black arrows – non-perfused vessel-like structures, White arrows – perfused vessels, Red arrows – human CD31 positive vessel-like structures, Blue arrows – mouse CD31 positive vessel-like structures, and Black dotted line – the boundary between Matrigel plug and mouse tissue. **p < 0.01, and ***p < 0.001. Scale bar: 20 × = 100 μm, 50 × = 50 μm.

Matrigel plugs with HIF-1α silenced SHED showed delayed vessel formation and perfusion compared with that of normal SHED plugs. In control groups, either siControl or CTR, vessel-like structures (Figures 3A,C, black arrows) were seen after 3 days while perfused vessels (white arrows) were observed after 7 days of implantation. Similar structures, however, could not be seen either in siHIF-1α or YC-1 group. The quantification of the number of non-perfused vessels and perfused vessels at 7 day samples showed that the silencing or chemically inhibition of HIF-1α in SHED significantly reduced the vascularization of the Matrigel plugs.

Immunohistochemistry staining for human CD31 revealed that the siControl and CTR groups contained more positive cells and vessel-like structures (Figures 3A,C, red arrows) compared with siHIF-1α or YC-1 group, indicating endothelial differentiation of HIF-1α intact SHED. The quantified percentages of human CD31 positive area at 7 days (Figures 3B,D) showed both siHIF-1α and YC-1 groups failed to induce a prominent vasculogenic response. Similarly, IHC for mouse CD31 disclosed that in 7 day samples, the siControl and CTR groups contained mouse CD31 positive vessel-like structures (Figures 3A,C, blue arrows). The black dotted line showed the boundary between Matrigel plug and mouse tissue. Mouse CD31 positive vessels were scarcely found in both siHIF-1α and YC-1 groups, which could be attributed to the indispensable role of HIF-1α expression in the induction of host blood vessel ingrowth into the implant.

### Silencing of HIF-1α in SHED Affects ROS Homeostasis and Cell Viability Under Stress *in vitro*

**FIGURE 4:**
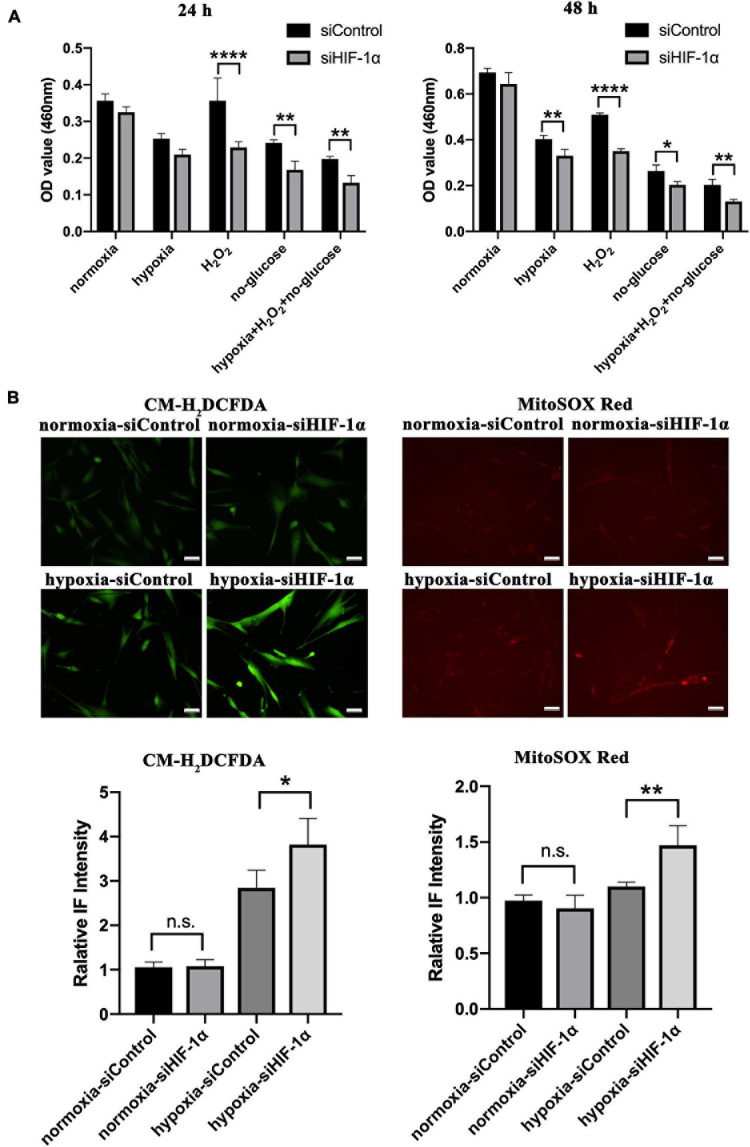
Silencing HIF-1α in SHED limits their cell viability and ROS homeostasis under stress *in vitro.* **(A)** CCK-8 assay of siControl and siHIF-1α SHED in normoxia, hypoxia, H_2_O_2_, glucose deprived medium, and hypoxia + H_2_O_2_ + glucose-deprived medium. **(B)** Immunofluorescence for CM-H_2_DCFDA (green) and MitoSOX (red) showed the cytoplasmic and mitochondrial ROS levels in SHED, respectively. *p < 0.05, **p < 0.01, and ****p < 0.0001. Scale bar = 50 μm.

Having observed a significantly reduced cellularity in HIF-1α silenced SHED in Matrigel plugs *in vivo*, we aimed to examine the role of HIF-1α signaling in cell survival under different stress conditions *in vitro*. CCK-8 assay results (Figure 4A) revealed that there is no significant difference in cell viability between siControl and siHIF-1α SHED in normal culture conditions. In hypoxia condition, cell survival was reduced in both groups compared with that of normoxia while siHIF-1α SHED showed a significant reduction than siControl after 48 h of culture. Furthermore, HIF-1α seems to play a more important role in maintaining cell viability under oxidative stress and glucose-deprived conditions as shown by significantly reduced OD values in siHIF-1α SHED compared with that of siControl group at both 24 and 48 h.

Additionally, cytoplasmic and mitochondrial ROS levels in SHED were evaluated by using CM-H_2_DCFDA (green, Figure 4B) and MitoSOX (Red, Figure 4B), respectively. The results showed increased cytoplasmic ROS levels in cells exposed to hypoxia while the levels were significantly higher in HIF-1α silenced SHED (*p* < 0.05). Similar results were observed by mitochondrial superoxide indicator, which showed higher oxidative stress in the mitochondria of siHIF-1α SHED in hypoxia (*p* < 0.01) compared with that of siControl SHED.

### Silencing of HIF-1α Suppressed the Expression of Proteins Important in Metabolic Adaptations *in vitro*

**FIGURE 5:**
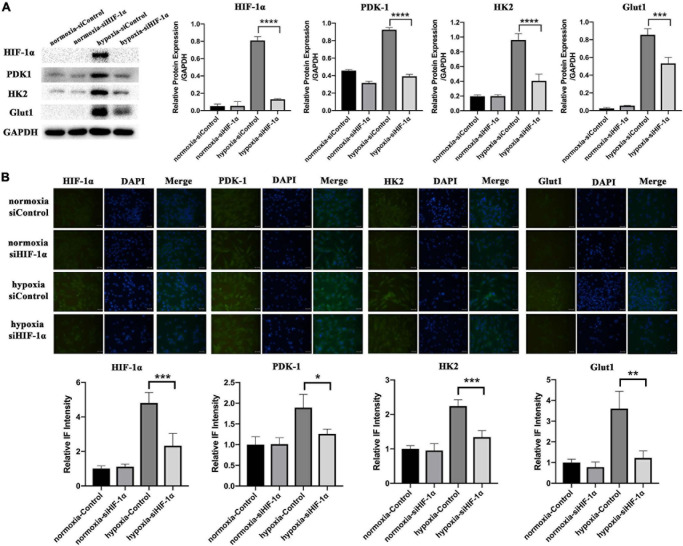
Silencing of HIF-1α suppressed the expression of proteins important in metabolic adaptations *in vitro*. **(A)** Protein levels of HIF-1α, PDK1, HK2, and Glut1 in siControl and siHIF-1α SHED in normoxia and hypoxia as shown by western blotting. **(B)** Immunofluorescence and relative intensity quantification for HIF-1α, PDK1, HK2, and Glut1 in siControl and siHIF-1α SHED in normoxia and hypoxia *p < 0.05, **p < 0.01, ***p < 0.001, and ****p < 0.0001. Scale bar = 100 μm.

Similar to *in vivo* assays, we also examined the levels of PDK1, HK2, and Glut1 proteins *in vitro* cell cultures by WB (Figure 5A) and IF (Figure 5B). There was not much difference in PDK1, HK2, and Glut1 protein expressions between the two cell groups in normoxia. In contrast, the three proteins were significantly activated in siControl SHED under hypoxia, while markedly impaired in HIF-1α silenced SHED. These results confirmed the role of HIF-1α in regulating the expression of genes responsible for metabolic adaptations under oxidative stress in SHED.

### Silencing of HIF-1α in SHED Reduced VEGF Secretion and Paracrine Effects on HUVECs

**FIGURE 6:**
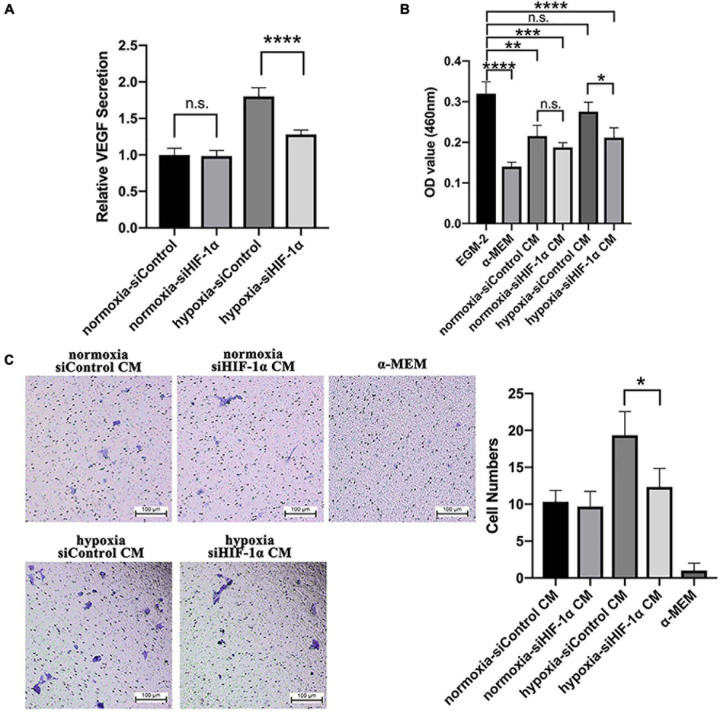
Silencing of HIF-1α in SHED reduced VEGF secretion and paracrine effects on HUVECs. **(A)** VEGF levels in different conditioned media as detected by ELISA and normalized to that of siControl under normoxia. **(B)** CCK-8 assay of HUVECs cultured in different conditioned media, EGM-2 (positive control), α-MEM (negative control). **(C)** *Trans*-well assay of HUVEC migration under different conditioned media, α-MEM (negative control). *p < 0.05, **p < 0.01, ***p < 0.001, and ****p < 0.0001. Scale bar = 100 μm.

The *in vivo* Matrigel plug assay results demonstrated significantly less mouse and human CD31 positive vessels and perfused vessels in siHIF-1α and YC-1 SHED groups, which indicated a crucial role of HIF-1α in mediating autocrine and paracrine angiogenic effects. Therefore, we examined the levels of VEGF protein, the most important angiogenic factor, in the conditioned media collected from siControl and siHIF-1α SHED by ELISA. The results (Figure 6A) revealed that siHIF-1α SHED had secreted significantly lower VEGF levels than siControl SHED in hypoxia (*p* < 0.0001). When we used the conditioned media to culture HUVECs, CCK-8 assay results (Figure 6B) showed that HUVECs cultured in conditioned medium from siControl SHED under hypoxia had the highest growth among the four conditioned media, which were all significantly higher than that of cultured in α-MEM. In contrast, conditioned medium collected from siHIF-1α SHED under hypoxia failed to show increased proliferation of HUVECs than siControl SHED conditioned medium in normoxia.

*Trans*-well assay was performed to evaluate the function of HIF-1α expression in SHED on inducing migration of HUVECs. The representative images and quantification of migrated cells (Figure 6C) revealed that conditioned medium from siControl SHED could significantly enhance the migration of HUVECs while conditioned medium from HIF-1α silenced SHED failed to do so. Collectively, the results confirmed that HIF-1α expression plays a crucial role in mediating paracrine angiogenic effects of SHED under hypoxia.

## Discussion

The harsh microenvironment, which could be due to hypoxia, low nutrients, and oxidative stress, is the main reason for low post-implantation stem cell survival. Physiologically, the HIF-1α signaling pathway is reported to be activated to counter hypoxic conditions. In the current study, we uncovered dual functions of HIF-1α in dental stem cells as adaptive responses to the microenvironmental stress. Firstly, HIF-1α is essential to activate the downstream genes responsible for switching from oxidative phosphorylation to glycolysis in order to maintain redox balance at the early stage of cell adaptation. Secondly, HIF-1α dependent VEGF secretion plays an essential role in inducing the angiogenic response by promoting the proliferation and recruitment of host vascular endothelial cells and differentiation of dental stem cells into endothelial cells.

In the early stage of cell implantation, there is almost no vessel invasion from the host since it is an extremely slow process (Stegen et al., 2016). Therefore, it is inevitable that the stem cell niche gets devoid of oxygen and nutrients. In normal oxygen levels, ROS, mainly produced by mitochondria, regulates cell proliferation and angiogenesis. In hypoxia, however, the excessively increased ROS production can destroy cell membranes (Sun et al., 2019), damage stem cells’ self-renewal capacity, and induce cell apoptosis (Zhou et al., 2014). Stem cells are always more vulnerable to the destructive impacts of drastic increase in ROS than other differentiated cells. In our study, we observed that there was a significant hypoxic environment particularly in the center of Matrigel plugs and markedly decreased cell numbers consistent with the low oxygen tension *in vivo*. This finding indicated the crucial role of HIF-1α in maintaining the viability of SHED under hypoxia.

Reactive oxygen species and HIF-1α signaling have been known to be involved in various diseases, such as tumors and ischemia injury. It was reported that PDK1 is a direct target of HIF-1α and plays an essential role in mitochondrial ROS production, maintenance of ATP levels, and adaptation to hypoxia (Kim et al., 2006). PDK1 inhibits mitochondrial respiration and ROS production by restraining pyruvate metabolism via the TCA cycle. Similarly, in our results, we observed that the HIF-1α silenced cells in hypoxia failed to activate PDK1, which is reflected in higher cellular and mitochondrial ROS levels observed in these cells that is also associated with lower cell survival. Moreover, it has been recently found that HIF-1α can be translocated to the mitochondria in response to hypoxia or H_2_O_2_ directly through a PDK1 independent manner and prevent oxidative stress-related apoptosis (Li et al., 2019).

In order to maintain a sufficient energy supply while maintaining a low oxidative stress, cells in hypoxia are forced to switch from oxidative phosphorylation to less efficient glycolytic pathway, which requires more glucose uptake into the cells (Polet and Feron, 2013). HIF-1α is considered a primary oxygen sensor and a critical transcriptional regulator of numerous genes involved in the glycolysis, the process that consumes glucose (Moldogazieva et al., 2020). In this process, HIF-1α promotes the expression of Glut1, which is highly abundant in membranes of mammalian cells and facilitates glucose transport (Denko, 2008; Yao et al., 2018). Similarly, in our results, Glut1 was highly activated in SHED under hypoxia. Once HIF-1α was silenced or inhibited, Glut1 expression was affected in hypoxia simultaneously. On the other hand, intake of more glucose alone is far from enough for the cell survival due to the inhibitory effects of downstream products of the glycolytic process, such as glucose-6-phosphate (G-6-P). As the first rate-limiting enzyme, hexokinase (HK) catalyzes the conversion of glucose to G-6-P (Lis et al., 2016). Among all four isoforms of HK (HK1, HK2, HK3, and HK4), HK2 was found to be highly expressed in patients with carcinomas (DeWaal et al., 2018) and considered the most efficient isoform in aerobic glycolysis (Gong et al., 2012). After interacting and binding to one specific protein in the outer membrane of mitochondria, HK2 acts in not only enhancing the glycolysis process but also facilitating ATP production through activation of ATP synthesis-related enzymes (Xu and Herschman, 2019). Once HK2 is deleted, the glycolytic flux and the proliferation of cancer cells have been inhibited (DeWaal et al., 2018) with increased susceptibility to apoptosis induced by hypoxia (Wolf et al., 2011). Moreover, it has been demonstrated that HIF-1α is also involved in several additional signaling pathways that lead to accelerated glycolytic levels, such as PI3K/Akt/mTOR, Raf/MAPK, and AMPK (DeBerardinis et al., 2008).

Our previous study demonstrated that HIF-1α stabilization in SHED under normoxia up-regulated the VEGF expression, which resulted in increased endothelial differentiation via autocrine effects and elevated angiogenesis via paracrine signaling both *in vitro* and *in vivo* (Han et al., 2020a). Accordingly, it has been shown that VEGF expression was significantly down-regulated when HIF-1α was knocked down in bone marrow stem cells and cancer cells (Chen et al., 2016; Zhang et al., 2018), leading to poorly vascularized constructs (Stegen et al., 2016). HIF-1α/VEGF signaling pathway activation promoted the survival, proliferation, and migration of endothelial cells, which are vital steps in blood vessel formation (Yang et al., 2016; Rattner et al., 2019). The results of the current study revealed that the reduced VEGF secretion following HIF-1α silencing in SHED led to diminished proliferation and migration of HUVECs as well as vascularization in accordance with the previously reported findings in relation to other types of cells. Moreover, upregulation of VEGF via HIF-1α activation under hypoxia can also protect cardiomyocytes against ischemia injury (Dai et al., 2007) and repair ischemia/reperfusion injury in lung tissue *in vivo* (Fan et al., 2019).

Taken together, our data indicate that HIF-1α serves as a central regulator of redox homeostasis and glucose metabolism in maintaining cell survival at the early stages of hypoxia. In securing long-term cell survival, HIF-1α also plays an essential role in inducing an angio-/vasculogenic response via VEGF-induced differentiation, proliferation and migration of endothelial cells. However, the exact contribution of each of these factors and the involvement of other signaling pathways are yet to be unraveled. In addition, growth factors other than VEGF might also be involved in the angio-/vasculogenic process. Further investigations need to be done to elucidate the complex crosstalk between redox homeostasis, reprogrammed metabolism, cell survival, and angio-/vasculogenesis.

In conclusion, our results demonstrated that endogenous HIF-1α signaling is crucial to dental stem cell-based tissue regeneration. HIF-1α induced ROS homeostasis and glycolysis-related gene expression are involved in the early stage of post-implantation survival of SHED, while VEGF is primarily responsible for the angio-/vasculogenic response. Based on these pivotal roles, HIF-1α could be used as a highly promising target for improving post-implantation cell survival and developing new treatment approaches in stem cell-based tissue engineering.

## Data Availability Statement

The raw data supporting the conclusions of this article will be made available by the authors, without undue reservation.

## Ethics Statement

The animal study was reviewed and approved by Committee on the Use of Live Animals in Teaching and Research, The University of Hong Kong.

## Author Contributions

YH and WD conceived and designed the project. YH, QC, and LZ participated in designing the experiments, analyzing data, and assembling figures. YH and WD contributed to drafting, editing, and revising the manuscript. All authors contributed to the article and approved the submitted version.

## Conflict of Interest

The authors declare that the research was conducted in the absence of any commercial or financial relationships that could be construed as a potential conflict of interest.

## Publisher’s Note

All claims expressed in this article are solely those of the authors and do not necessarily represent those of their affiliated organizations, or those of the publisher, the editors and the reviewers. Any product that may be evaluated in this article, or claim that may be made by its manufacturer, is not guaranteed or endorsed by the publisher.
